# Molecular Detection and Prevalence of *Coxiella burnetii* in Ticks from Namibia: A Regional and Genus-Specific Analysis

**DOI:** 10.3390/pathogens14121262

**Published:** 2025-12-10

**Authors:** Pricilla Mbiri, Walter Muleya, Enos Moyo, Alaster Samkange, Ophelia Chuma Matomola, Vonai Charamba, Urban Ujava, Elfriede Esmerelda Hoebes, Frank Chitate, Foibe Waalukeni Tuyenikelao Neshindo, Joseph Kapapero, Christian Winter, Sabrina Weiss, Emmanuel Nepolo, Lillian Pazvakawambwa, Simbarashe Chitanga

**Affiliations:** 1 Department of Production Animal Clinical Studies, School of Veterinary Medicine, University of Namibia, Windhoek 13301, Namibia; asamkange@unam.na; 2 Department of Biomedical Sciences, Samora Machel School of Veterinary Medicine, University of Zambia, Lusaka 10101, Zambia; 3 Department of Public Health Medicine, School of Nursing and Public Health, College of Health Sciences, University of KwaZulu-Natal, Durban 4000, South Africa; moyoenos@yahoo.co.uk; 4 Department of Preclinical Studies, School of Veterinary Medicine, University of Namibia, Windhoek 13301, Namibia; 5 Department of Animal Production, Agribusiness and Economics, Faculty of Agriculture, Engineering and Natural Sciences, University of Namibia, Windhoek 13301, Namibia; 6 Department of Paraclinical Studies, School of Veterinary Medicine, University of Namibia, Windhoek 13301, Namibia; fchitate@unam.na; 7 Directorate of Veterinary Services, Ministry of Agriculture, Water and Land Reform, Windhoek 9000, Namibia; 8 Robert Koch Institute, Centre for International Health Protection, 13353 Berlin, Germany; 9 Department of Human Biology and Translational Medicine, School of Medicine, University of Namibia, Windhoek 13301, Namibia; 10 Department of Computing, Mathematical and Statistical Sciences, University of Namibia, Windhoek 13301, Namibia; 11 Department of Biomedical Sciences, School of Health Sciences, University of Zambia, Lusaka 10101, Zambia; 12 Department of Animal Sciences, Faculty of Animal and Veterinary Sciences, Botswana University of Agriculture and Natural Resources, Gaborone 999077, Botswana

**Keywords:** *Coxiella burnetii*, ticks, Namibia, One Health, molecular epidemiology

## Abstract

*Coxiella burnetii* (*C. burnetii*) is a zoonotic pathogen with significant public and veterinary significance. Whilst livestock are considered as primary reservoirs of the pathogen, ticks play a crucial role in transmission and environmental contamination. Within Namibia, there is serological evidence of pathogen circulation in livestock and wildlife. However, no study has ever been conducted to determine the prevalence of *C. burnetii* in ticks in Namibia. Thus, this study investigated the prevalence and genetic diversity of *C. burnetii* in ticks collected from two different ecological settings. A total of 502 ticks (*Rhipicephalus*, *Amblyomma*, and *Hyalomma*) collected from 278 cattle (139 from each of the tropical Zambezi and arid Khomas regions) were screened for *C. burnetii* using PCR targeting the genus-specific *16S rRNA* and the species-specific isocitrate dehydrogenase (*icd*) genes. Based on the isocitrate dehydrogenase (*icd*) genes, an overall prevalence of 8% (40/502) was observed for *C. burnetii*, with significantly higher infection rates observed in the more tropical Zambezi region (11.7%) when compared to the more arid Khomas region (2.8%) [*p* = 0.0005]. Variation was observed amongst tick species [*p* = 0.00121], with prevalence being slightly higher in *Amblyomma* ticks (12.9%) and *Hyalomma* (10.6%) as compared to *Rhipicephalus* ticks (3.6%). Phylogenetic analysis based on the *icd* gene sequences confirmed 99–100% identity with *C. burnetii* strains from around the world, thus confirming the circulation of this pathogen in ticks, ultimately supporting their potential role in the epidemiology of this pathogen in Namibia. The observed regional prevalence difference could be driven by variation in the ecological factors, with the subtropical climatic conditions of Zambezi likely favoring higher tick infection rates. Our findings highlight the need for One Health–based surveillance to mitigate the risks associated with pathogen risk. This study provides the first molecular evidence of *C. burnetii* in ticks in Namibia, highlighting their role in the pathogen’s epidemiology and providing relevant information for informed control strategies.

## 1. Introduction

*Coxiella burnetii* (*C. burnetii*), the causative agent of Q fever, is a globally significant zoonotic pathogen with the ability to infect both animal and human populations [1]. Livestock are considered the primary reservoirs, with infections often asymptomatic and at times presenting with reproductive disorders, including abortions, stillbirths, and infertility [2]. As such, clinical infections in livestock are associated with significant economic losses. Infected animals contaminate the environment through aborted fetuses and infected placentas, which can lead to airborne dissemination of the bacteria, resulting in infections in humans in close contact with the animals, explaining the increased risk of infection in individuals involved with livestock management [1,3]. Besides the inhalation of contaminated aerosols, other routes of transmission to humans include the consumption of unpasteurized dairy products or direct contact with infected animals [4]. Within humans, Q fever may present as either an asymptomatic infection or an acute febrile illness, with the potential to progress to severe chronic diseases, including endocarditis and pneumonia [5].

The association of *C. burnetii* with ticks has long been suspected, dating back to the first isolation of the bacterium from a tick [6]. Since then, the bacterium has been reported in several tick species [6]. Ticks play a crucial role as a reservoir of infection in the sylvatic cycle, while also serving as the primary agents responsible for introducing the pathogen into the domestic cycle [7]. Within the ticks themselves, maintenance of the pathogen has been shown to occur through transstadial and transovarial transmission [8], ensuring perpetual infection within and across generations, thus cementing their central role as reservoirs of the infection. Experimental studies indicate that fecal secretion is a potential pathway for environmental contamination and could serve as a source of infectious material for mammalian hosts, for instance, through aerosol transmission [9,10,11].

The molecular detection and characterization of *Coxiella* species, particularly distinguishing *C. burnetii* from Coxiella-like endosymbionts (CLEs), primarily rely on genetic markers such as the *16S rRNA* gene due to its combination of conserved and variable regions, which enable PCR amplification and phylogenetic analysis of *Coxiella burnetii* and related bacteria. [12,13]. The *icd* (isocitrate dehydrogenase) gene, a protein-coding housekeeping gene, offers greater sequence variation than *16S rRNA*, making it a more robust marker for distinguishing *C. burnetii* from *Coxiella*-like endosymbionts (CLEs) and for elucidating genetic diversity within the *Coxiella* genus [8].

Within Namibia, there have been reports of the circulation of *C. burnetii* based on serological surveys and studies performed in humans [14] and domestic animals and wildlife [15,16], indicating the circulation of this zoonotic pathogen in the mammalian hosts. However, the role that ticks play in the epidemiology of this pathogen has not been investigated in Namibia. Therefore, this study aimed to determine the prevalence and identify the species of *C. burnetti* present in ticks collected from livestock across different regions of Namibia using molecular techniques, as well as to determine the phylogenetic relationships of the obtained sequences with known *C. burnetti* groups.

## 2. Materials and Methods

### 2.1. Study Sites

**Figure 1:**
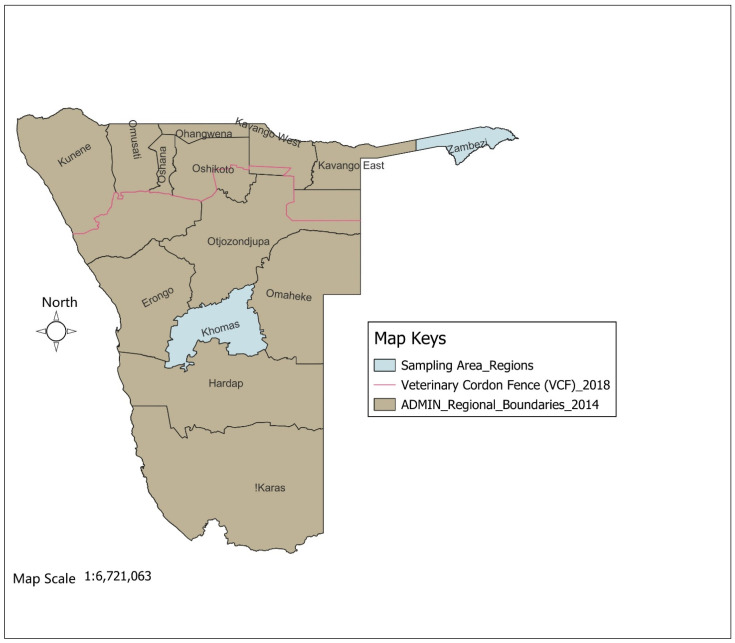
Map of Namibia showing the study regions (Khomas and Zambezi).

The study was conducted in the Zambezi and Khomas regions of Namibia (Figure 1), which were selected due to their distinct ecological and epidemiological characteristics. A total of 502 ticks were collected from 278 cattle (139 cattle per region) across two study areas, as determined using the Epitools sample size calculator [17] with an estimated 90% tick prevalence, 95% confidence level, and 5% precision. The ticks were collected across two seasons: winter (May–October) and summer (November–April). In the Khomas region, 212 ticks were collected from six commercial farms, and in the Zambezi region, 290 ticks were collected from cattle at 20 high-traffic crush pens. All specimens were preserved in 70% ethanol and transported to the laboratory for morphological identification using standard taxonomic keys [18].

#### Tick Identification

Individual ticks were surface-sterilized through three sequential washes in sterile deionized water. Genomic DNA was extracted using the Zymo Research Quick-DNA Miniprep Plus Kit (Irvine, CA, USA), which incorporates bead-beating lysis. Identification of the ticks was conducted morphologically using established keys [18] and through sequencing targeting the mitochondrial *12S rDNA* and *16S rDNA* genes [19]. PCR was used to amplify segments of the genome for *12S rDNA* and also *16S rDNA* using primers listed in Table 1. This was a 20 µL reaction consisting of 10 µL 2X one-taq master-mix, 0.48 µM of the forward and reverse primers, 5.08 µL of nuclease-free water, and 3 µL of the template DNA. The conditions used were initial denaturation at 95 °C for 1 min, denaturation at 95 °C for 30 s, annealing at the set primer temperature for 30 s, extension at 72 °C for 1 min, and final extension at 72 °C for 10 min. The reaction was run for 35 cycles and then held at 4 °C until further processing. Amplicons were visualized by gel electrophoresis with ethidium bromide staining.

### 2.2. Molecular Screening and Detection of Coxiella

*Coxiella burnetii* detection was performed using a validated two-step PCR protocol [20] with initial screening performed using primers (Table 2) targeting a 1450 bp fragment of the *16S rRNA* gene and subsequent confirmation performed using primers (Table 2) targeting a 900 bp fragment of the *C. burnetii-specific* isocitrate dehydrogenase (*icd*) gene using OneTaq^®^ Quick-Load 2X Master Mix (New England BioLabs, Ipswich, MA, USA). PCR conditions included: 98 °C for 3 min (initial denaturation), followed by 35 cycles of 98 °C for 15 s, 46 °C for 30 s, and 68 °C for 45 s, with a final extension at 68 °C for 5 min. Amplified products were electrophoresed on ethidium bromide-stained 1% agarose gels. Only samples with reproducible bands of expected sizes and clean negative controls were considered positive and selected for sequencing.

### 2.3. Statistical Analysis

Prevalence estimates and associated factors were calculated using EpiTools epidemiological calculators [17]. Associations between categorical variables (region, sex of tick, tick genus) and infection status were assessed using chi-square tests.

To further examine associations, a multiple logistic regression model in R was constructed. Adjusted odds ratios with 95% confidence intervals (CIs) were derived by exponentiating the regression coefficients, allowing for the quantification of the strength of the associations while controlling for potential confounding variables. All statistical tests were evaluated using a significance level of α = 0.05.

### 2.4. Sequencing of the 12S rDNA, 16S rDNA, 16s rRNA and the Icdtrg Genes

The library preparation process for positive PCR amplicons and control samples was conducted using SQK-RBK114-96 kits (Oxford Nanopore Technologies, Oxford, UK), following standardized protocols. PCR products underwent purification with AMPure^®^ XP Beads (Oxford Nanopore Technologies, Oxford, UK) at a 0.6:1 bead-to-sample volume ratio, processed in 96-well PCR plates according to established procedures. For barcoding, 7.5 µL of purified DNA from each sample was processed, with sample concentrations verified using Qubit 4 fluorometric quantification (Thermo Fisher Scientific, Waltham, MA, USA) with the 1×dsDNA HS assay (Thermo Fisher Scientific, Waltham, MA, USA). After normalization, the barcoded samples were pooled and subjected to additional purification with AMPure^®^ XP Beads at a 1:1 ratio, including two washes with 80% ethanol solution. Finally, the library was loaded onto the R10.3 nanopore flow cells and sequenced using the Oxford Nanopore MinION sequencing technology (Min-101B platform) (Oxford Nanopore Technologies, Oxford, UK).

### 2.5. Phylogenetic Analysis

Nanopore sequencing reads targeting the *C. burnetii* icd gene were processed using a custom bioinformatics workflow. Raw FASTQ reads were first trimmed to remove adapter and primer sequences using Cutadapt v4.9 [21].

Two forward primer sequences (5′-CGGAGTTAACCGGAGTATCCA-3′ and 5′-ATTGAAGAGTTTGATTCTGG-3′) and their corresponding reverse complements (CCGTGAATTTCATGATGTTACCTTT and CGGCCTCCCGAAGGTTAG) were specified for removal, allowing a maximum error rate of 15% and requiring a minimum overlap of 12 bp. Only reads with a minimum length of 200 bp were retained. Trimming was executed using six processing threads. Subsequently, quality filtering of the trimmed reads was performed using Filtlong v0.2.1 (https://github.com/rrwick/Filtlong, accessed on 10 October 2025), retaining the top 95% of reads based on quality and enforcing a minimum read length threshold of 200 bp. The resulting high-quality reads were then aligned to the *C. burnetii* reference genome (GenBank accession LC319607) using Minimap2 v2.28 [22] with the map-ont preset optimized for Oxford Nanopore Technologies (ONT) reads. The SAM alignment output was converted to BAM format, sorted, and indexed using SAMtools v1.21 [23] to facilitate downstream analysis. Consensus sequence polishing was carried out using Medaka v1.12.1 (https://github.com/nanoporetech/medaka, Oxford Nanopore Technologies, Oxford, UK, accessed on 10 October 2025), which applies neural network-based models to correct residual basecalling errors in ONT reads. The final consensus sequence was generated with the model r1041_e82_400bps_sup_v5.2.0 using four CPU threads and saved for downstream analysis.

Five (5) *16S rRNA* and eight (8) *icdtrg* gene sequences were successfully generated and deposited in Genbank under accession numbers PX401868–PX401872 and PX434716 –PX434723, respectively (Supplementary Table S1). These sequences were subsequently compared against the NCBI nucleotide collection using BLASTn version 2.17.0 for preliminary identification.

For phylogenetic reconstruction, relevant reference sequences were obtained from GenBank, and sequence alignment was performed using ClustalW (version 1.6) [24] in MEGA 12 [25]. Phylogenetic analysis was performed using the Tamura-3 model in MEGA 12, with bootstrap values obtained from 1000 replicates.

## 3. Results

### 3.1. Coxiella Screening and Identification

Initial screening using the genus-specific primers targeting the *16S rRNA* gene revealed an overall infection prevalence of 14.9% (75/502). Confirmatory PCR targeting the *C. burnetii-specific isocitrate dehydrogenase* (isocitrate dehydrogenase) gene was performed to validate the infection status. This confirmatory step established the overall confirmed prevalence of *C. burnetii* in ticks at 8% (40/502) (95% CI: 5.8–10.7%). There was a significant association between region and *Coxiella* prevalence (*p*-value = 0.0005), with ticks from Zambezi showing a significantly higher prevalence (11.7% (34/290); 95% CI: 8.3–16.0%) compared to those from Khomas (2.8% (6/212); 95% CI: 1.0–6.1%), with an odds ratio of 4.56 (95% CI: 1.88–11.07), indicating a markedly increased likelihood of infection in Zambezi (Table 3). While male ticks had a slightly higher prevalence (8.8%; 95% CI: 6.0–12.5%) than females (7.9%; 95% CI: 3.6–20.0%), this difference was not statistically significant (*p*-value > 0.05). Tick genera were significantly associated with Coxiella positivity (*p* = 0.0121), with *Amblyomma* exhibiting the highest prevalence (12.9%; 4/31; 95% CI: 3.6–29.8%] vs. *Rhipicephalus* (3.6%), followed by *Hyalomma* (10.6%; 29/274; 95% CI: 7.2–15.0%). A statistically significant difference was identified between the dry and wet seasons (*p* = 0.016). The prevalence during the dry season (11.9%) was significantly higher than in the wet season (5.8%), with an OR of 2.25 [95% CI: 1.17–4.31]. This finding suggests that the risk of *Coxiella* infection more than doubles during dry conditions, potentially due to increased environmental dust or animal congregation around limited water sources, which may enhance pathogen transmission.

**Figure 2:**
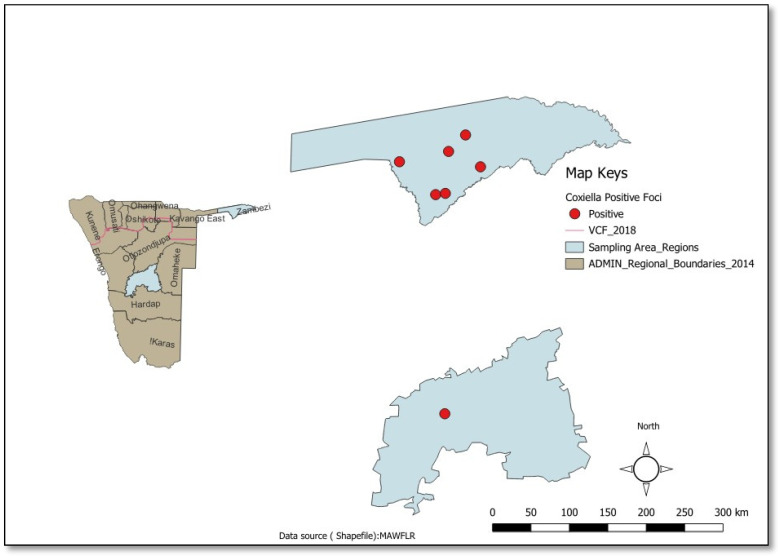
Spatial Distribution of *C. burnetii* (Khomas and Zambezi Regions, Namibia). The dot represents positive foci.

When considered by sampling region, the Zambezi had more positive foci (sampling points with one or more positive ticks) as compared to the Khomas region. This indicated that *C. burnetii*-infected ticks were more widespread in the Zambezi region as compared to the Khomas region [Figure 2].

Nucleotide BLAST (BLASTn) comparison of the *16S rRNA* and *icd* gene sequences is shown in Table 4 below.

**Figure 3:**
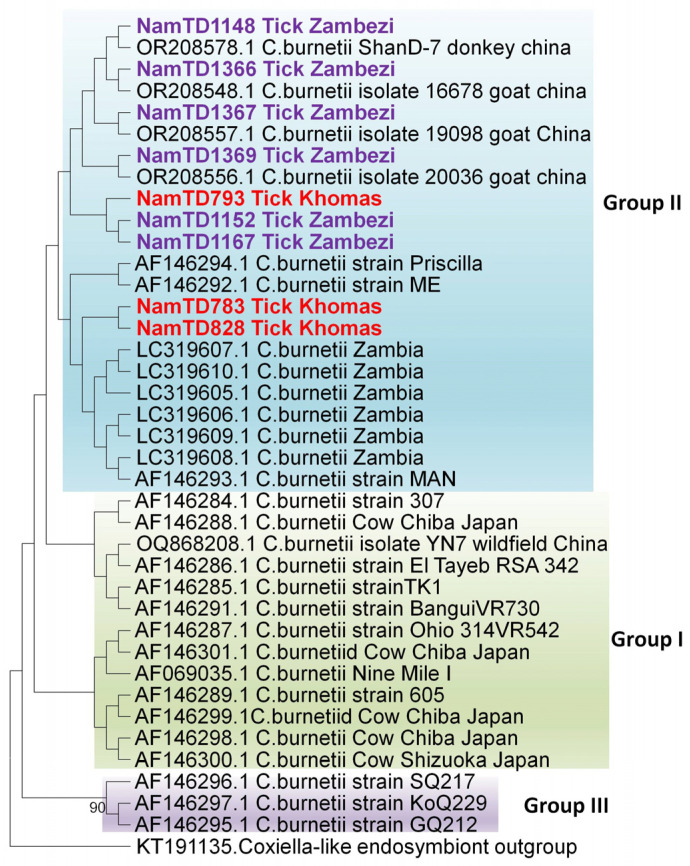
Evolutionary relationships of *Coxiella* strains based on 900 bp of the *icd* gene. The maximum likelihood tree was constructed using the Tamura-3 model with 1000 bootstrap replicates as a measure of confidence using the MEGA 12 software. Sequences derived from *Coxiella*-positive samples collected in this study are prefixed [“NamTD”] in the tree. Only bootstrap values more than 75% are shown at branch nodes.

Further, phylogenetic analysis (Figure 3) of the *icd* gene sequences (900 bp) revealed that *C. burnetii* isolates clustered into four distinct groups as reported by Nguyen & Hirai [26]. Group I comprised isolates from acute human Q fever cases (Japan & Central America), ticks (Egypt & USA), and cows with persistent infections (Japan & USA). Group II included isolates from chronic human Q fever patients (France), goats (USA), a dog (Zambia), and rodents (Zambia); all the Namibian samples clustered within this group. Group III consisted of isolates from chronic human Q fever cases reported in Canada and the USA.

### 3.2. Ticks Screening and Identification

**Figure 4:**
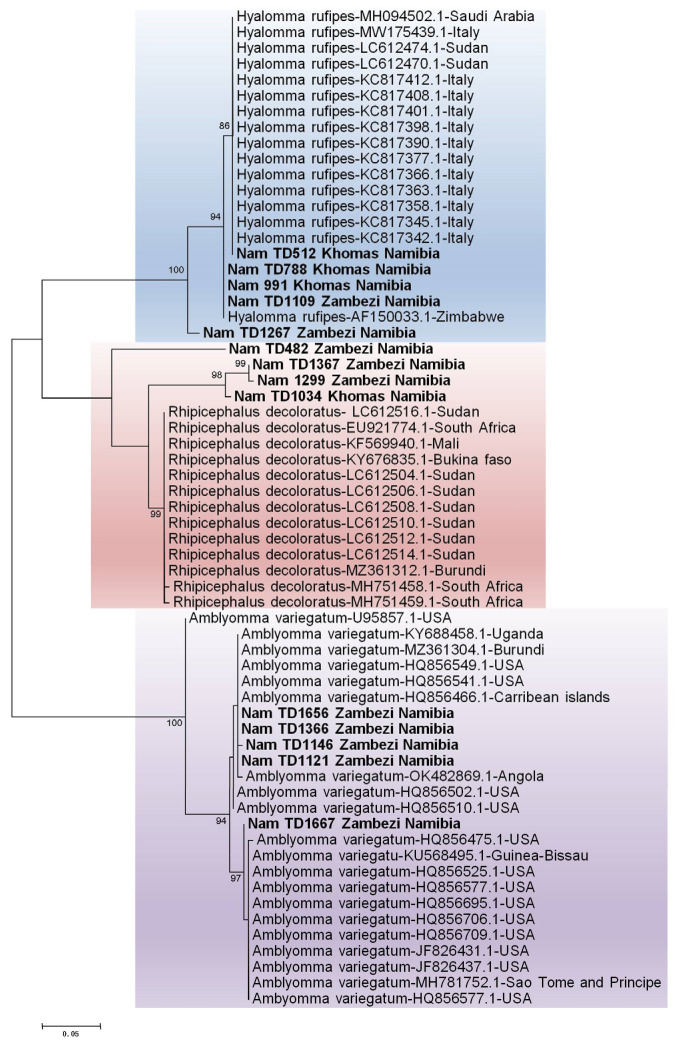
Identification of ticks based on the *12S rRNA* gene. The maximum likelihood tree was constructed using the Tamura-3 model with 1000 bootstrap replicates as a measure of confidence using the MEGA 12 software. Sequences derived from this study are prefixed [“NamTD”] in the tree.

**Figure 5:**
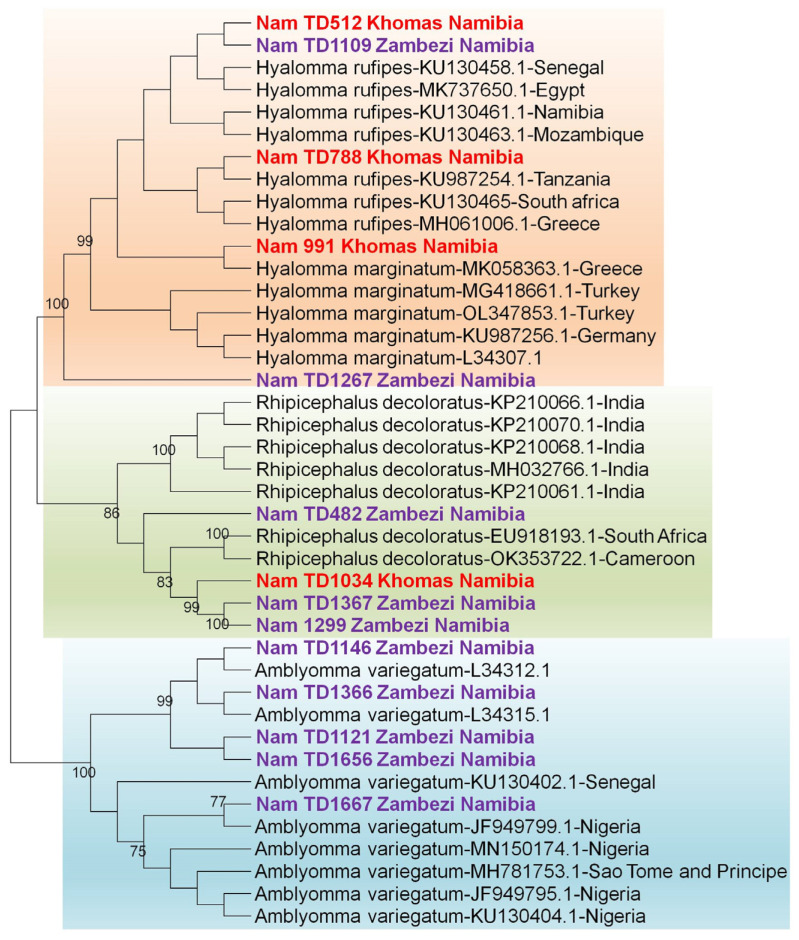
Identification of ticks based on the *16S rRNA* gene. The maximum likelihood tree was constructed using the Tamura-3 model with 1000 bootstrap replicates as a measure of confidence using the MEGA 12 software. Sequences derived from this study are prefixed [“NamTD”] in the tree.

Molecular identification of the ticks was performed by sequencing the *12S rRNA* and *16S rRNA* genes. While initial BLASTn vesion 2.17.0 results suggested identities for the tick samples [S1] with accession numbers PX442689-PX442702 based on the *16S rRNA* gene and PX443472-PX443485 based on the *12S rRNA* gene, a subsequent phylogenetic analysis was conducted to provide conclusive species assignment. The resulting phylogenetic tree clearly demonstrated that our sequences formed distinct, well-supported clades with verified reference sequences for *Amblyomma variegatum*, *Hyalomma rufipes*, *Hyalomma truncatum*, *Rhipicephalus evertsi evertsi*, *Rhipicephalus sanguineus*, and *Rhipicephalus evertsi mimeticus*, thereby confirming their taxonomic status. [Figure 4 and Figure 5].

Phylogenetic analysis confirmed the initial BLAST identifications, with all Namibian sequences clustering within well-supported clades for *Hyalomma rufipes*, *Rhipicephalus* species, and a distinct *Amblyomma variegatum*.

## 4. Discussion

Our study reveals the circulation of *C. burnetii* in various tick species collected from two regions of Namibia, contributing to the body of knowledge on the potential role of ticks in the epidemiology of *C. burnetii* in Africa [27,28,29,30,31,32,33,34,35]. The confirmation of our results using the isocitrate dehydrogenase gene allowed us to distinguish between *C. burnetii* and Coxiella-like endosymbionts, which are endosymbionts for ticks, serving as a potential source of vitamins [36] and playing a role in the fecundity of ticks [37]. Across different ticks collected from various hosts in Africa, the reported prevalence of the pathogenic *C. burnetii* ranges from as low as 2.9% to as high as 41% [36]; our findings fall within the same range. However, some studies conducted with ticks from wild and domestic animals have not been able to show the presence of the pathogen in ticks within the region [38,39]. In general, ticks collected from domestic pets tend to have a higher prevalence [25,26,39] compared to those from livestock [28,35,40] and the environment [28].

It should be noted that a number of the reports emerging, especially in Africa, are based on engorged and/or semi-engorged ticks collected from hosts; thus, it cannot be discounted that the detected DNA is due to a pathogen in the host’s blood. Whilst our study does not prove the vector role of ticks in the epidemiology of *C. burnetii*, we can infer that the tick species in our study can be important as carriers/reservoirs of the pathogen, as has been previously reported in studies designed in the same way as ours [41]. However, the vector role of ticks for *C. burnetii* has long been established, with some recent studies showing that transmission within the tick can occur through transstadial and transovarial transmission [8,27,42]. Transmission of *C. burnetii* by ticks is not only through tick bites, with reports of high bacterial excretions in fecal material, which serve as a potential source of aerosol transmission [9,43]. Thus, the ticks in our study could still play a significant role in environmental pathogen circulation in this context. This is especially important, considering that pathogens excreted by ticks into the environment could adhere to particulate matter [44]; thus, an excretion could result in significant transmission, considering its potential persistence in the environment for long periods [45].

In the current study, *C. burnetii* was reported in all the ticks screened (*Hyalomma*, *Rhipicephalus*, and *Amblyomma*), with higher prevalence in the Amblyomma and Hyalomma species. Globally, over 40 species of ticks have been reported to contain DNA of *C. burnetii* [31] with variations in prevalence among tick species, as observed in our own study. The study by Eneku et al. [28] showed species variation, with higher prevalence reported in *Haemaphysallis* and *Rhipicephalus appendiculatus* when compared to *Amblyomma variegatum* and *Rhipicephalus decoloratus*. Notably, our study found no significant sex-based differences in prevalence, contrasting with some earlier reports [2], possibly suggesting that biological factors specific to *C. burnetii*, such as vertical (transovarial) transmission, may override typical sex-biased pathogen acquisition patterns, affecting both sexes equally depending on species and environmental context [46]. Alternatively, the lack of disparity could imply that environmental exposure (e.g., co-feeding or contact with infected habitats) plays a more critical role than host-seeking behavior in determining infection prevalence. These findings are crucial for understanding the long-term persistence of *C. burnetii* in tick populations, as they suggest that both male and female ticks may contribute equally to maintaining the pathogen in the natural environment.

Our study showed regional variation in the prevalence of infection in ticks, a finding which has also been previously reported [35]. These differences likely reflect distinct ecological settings, as parameters such as climate and vegetation are known to influence the distribution of pathogens [47,48,49]. For instance, the Zambezi region’s subtropical climate, characterized by high humidity and dense vegetation, contrasts sharply with the arid to semi-arid conditions of the Khomas region, where temperatures are higher and vegetation is sparse. This ecological divergence may explain the higher prevalence observed in Zambezi compared to Khomas. However, while environments with wet vegetation, such as the Zambezi, favor bacterial survival in the environment, they may paradoxically reduce the risks of aerosol transmission to mammalian hosts [49]. Notably, our findings also revealed a significant seasonal pattern, with the prevalence of *C. burnetii* being higher in ticks sampled during the dry season when compared to those sampled during the wet season. Such seasonal variation in the prevalence of tick-borne pathogens in ticks has been previously reported [49], indicating that some environmental factors may influence pathogen acquisition and/or persistence in the ticks. In our context, we speculate that the increased prevalence of *C. burnetii* in the sampled ticks may be linked to the increased prevalence of infection in cattle hosts during dry periods, as their immunity wanes from environmental stress associated with reduced pasture availability, suggesting that certain environmental factors may influence pathogen acquisition and/or persistence in ticks. This highlights the need for further research into the prevalence of mammalian infections in these regions to assess potential disparities in infection dynamics.

## 5. Conclusions

This study highlights the critical role of ecological and climatic factors in driving *C. burnetii* transmission in Namibia. The higher prevalence of *C. burnetii* in ticks in Zambezi, particularly during the dry seasons, underscores the need for region-specific control measures. The lack of sex-based differences suggests that infection dynamics may be driven more by ecological and symbiotic factors (tick’s internal microbiome) than by tick behavior. Implementing integrated surveillance under a One Health framework will be vital in mitigating zoonotic risks and spillover.

## Figures and Tables

**Table 1:** Primers for tick species identification.

Gene	Forward 5′→ 3′	Reverse 5′ → 3′	Annealing Temperature
*16S rDNA*	CTGCTCAATGATTTTTTAAATGCTGT	GTCTGAACTCAGATCA AGT	48 °C
*12S rDNA*	AAACTAGGGATTAGATACCCT	AATGAGAGCGACGGGCGATGT	50 °C

**Table 2:** List of primer names and sequences used in this study.

Primer Name	Primer Sequence	Gene	Band Size
*icdtrg F*	CGGAGTTAACCGGAGTATCCA	*Icdtrg* ^1^	900 bp
*icdtrg R*	CCGTGAATTTCATGATGTTACCTTT		
*Cox 16S F*	ATTGAAGAGTTTGATTCTGG	*16S* ^2^	1450 bp
*Cox 16S R*	CGGCCTCCCGAAGGTTAG		

^1^ Isocitrate dehydrogenase gene, ^2^ 16S rRNA gene.

**Table 3:** Summary of Factors Associated with *Coxiella* Infection in Ticks.

Categorial Value	Level	Ticks Tested (n)	Positive Ticks (n)	Prevalence (%) [95% CI]	Odds Ratio [95% CI]	*p*-Value
Region	Zambezi	290	34	11.7 [8.3–16]	4.56 [1.9–11.1]	0.0005
	Khomas	212	6	2.8 [1–6.1]	Reference	
Tick genus	Amblyomma	31	4	12.9 [3.6–29.8]	4 [1.1–14.6]	0.0121
	Hyalomma	274	29	10.6 [7.2–15]	3.2 [1.4–7.5]	
	Rhipicephalus	197	7	3.6 [1.5–7.3]	Reference	
Tick sex	Male	375	28	7.5 [4.8–10.1]	0.8 [0.4–1.6]	0.5
	Female	127	12	9.5 [4.4–14.5]	Reference	
Season	Dry	269	32	11.9 [8–15.8]	2.3 [1.2–4.3]	0.016
	Wet	233	8	5.8 [2.8–8.8]	Reference	

**Table 4:** Table of sequence identity for samples sequenced in this study.

		Isocitrate Dehydrogenase Gene					16S Ribosomal RNA Gene			
	BLAST Result	Accession Number	% Identity	E Value	Fragment Length (bp)	BLAST Result	Accession Number	% Identity	E Value	Fragment Length (bp)
Nam_TD783	*C. burnetii*	AF146293	99.32	0.0	1300	*Uncultured Coxiella species*	OP411004	99.93	0.0	1545
Nam_TD793	*C. burnetii*	AF146293	99.86	0.0	1300					
Nam_TD828	*C. burnetii*	AF146293	99.86	0.0	1300					
Nam_TD1148	*C. burnetii*	AF146293	99.59	0.0	1300	*C. burnetii*	OQ152499	99.86	0.0	1444
Nam_TD1152	*C. burnetii*	AF146286	99.46	0.0	1301	*C. burnetii*	NR104916	100	0.0	1465
Nam_TD1167	*C. burnetii*	AF146286	99.46	0.0	1301	*C. burnetii*	NR104916	100	0.0	1465
NamTD1366	*C. burnetii*	AF146286	99.73	0.0	1301					
Nam_TD1367	*C. burnetii*	OR208578.1	99.57	0.0	711					
Nam_TD1369						*Uncultured Coxiella species*	OP411004	99.71	0.0	1545

## Data Availability

The sequences generated from this study are publicly available on GenBank.

## Supplementary Materials

The following supporting information can be downloaded at: https://www.mdpi.com/article/10.3390/pathogens14121262/s1, Table S1: Table of sequence identity for tick samples used in this study.

## References

[1] Maurin M., Raoult D. (1999). Q fever. Clin. Microbiol. Rev..

[2] Guatteo R., Seegers H., Taurel A.F., Joly A., Beaudeau F. (2011). Prevalence of *Coxiella burnetii* infection in domestic ruminants: A critical review. Veter–Microbiol..

[3] Cutler S.J., Bouzid M., Cutler R.R. (2007). Q fever. J. Infect..

[4] Porter S.R., Czaplicki G., Mainil J., Guattéo R., Saegerman C. (2011). Q Fever: Current State of Knowledge and Perspectives of Research of a Neglected Zoonosis. Int. J. Microbiol..

[5] Eldin C., Mélenotte C., Mediannikov O., Ghigo E., Million M., Edouard S., Mege J.L., Maurin M., Raoult D. (2017). From Q Fever to *Coxiella burnetii* Infection: A Paradigm Change. Clin. Microbiol. Rev..

[6] Duron O., Sidi-Boumedine K., Rousset E., Moutailler S., Jourdain E. (2015). The Importance of Ticks in Q Fever Transmission: What Has (and Has Not) Been Demonstrated?. Trends Parasitol..

[7] Hirai K., To H. (1998). Advances in the understanding of *Coxiella burnetii* infection in Japan. J. Vet. Med. Sci..

[8] Enferadi A., Sarani S., Mohammadipour S., Hasani S.J., Ajdari A., Asl M.N., Khademi P. (2024). Molecular detection of *Coxiella burnetii* in ticks collected from Iran. Infect. Genet. Evol..

[9] Körner S., Makert G.R., Mertens-Scholz K., Henning K., Pfeffer M., Starke A., Nijhof A.M., Ulbert S. (2020). Uptake and fecal excretion of *Coxiella burnetii* by Ixodes ricinus and Dermacentor marginatus ticks. Parasit. Vectors.

[10] Körner S., Makert G.R., Ulbert S., Pfeffer M., Mertens-Scholz K. (2021). The Prevalence of *Coxiella burnetii* in Hard Ticks in Europe and Their Role in Q Fever Transmission Revisited-A Systematic Review. Front. Vet. Sci..

[11] Celina S.S., Cerný J. (2022). *Coxiella burnetii* in ticks, livestock, pets and wildlife: A mini-review. Front. Vet. Sci..

[12] McLaughlin H.P., Cherney B., Hakovirta J.R., Priestley R.A., Conley A., Carter A., Hodge D., Pillai S.P., Weigel L.M., Kersh G.J. (2017). Phylogenetic inference of *Coxiella burnetii* by 16S rRNA gene sequencing. PLoS ONE.

[13] Stein A., Saunders N.A., Taylor A.G., Raoult D. (1993). Phylogenic homogeneity of *Coxiella burnetii* strains as determinated by 16S ribosomal RNA sequencing. FEMS Microbiol. Lett..

[14] Noden B.H., Tshavuka F.I., van der Colf B.E., Chipare I., Wilkinson R. (2014). Exposure and risk factors to *Coxiella burnetii*, spotted fever group and typhus group Rickettsiae, and Bartonella henselae among volunteer blood donors in Namibia. PLoS ONE.

[15] Cossu C.A., Ochai S.O., Troskie M., Hartmann A., Godfroid J., de Klerk L.M., Turner W., Kamath P., van Schalkwyk O.L., Cassini R. (2024). Detection of Tick-Borne Pathogen Coinfections and Coexposures to Foot-and-Mouth Disease, Brucellosis, and Q Fever in Selected Wildlife From Kruger National Park, South Africa, and Etosha National Park, Namibia. Transbound. Emerg. Dis..

[16] Samkange A., van der Westhuizen J., Voigts A.S., Chitate F., Kaatura I., Khaiseb S., Hikufe E.H., Kabajani J., Bishi A.S., Mbiri P. (2022). Investigation of the outbreaks of abortions and orchitis in livestock in Namibia during 2016-2018. Trop. Anim. Health Prod..

[17] Sergeant E.S.G. EpiTools Epidemiological Calculators. Ausvet, 2018. https://epitools.ausvet.com.au/static/Important-formulae-for-surveillance.pdf.

[18] Walker A.R., Bouattour A., Camicas J.L., Estrada-Pena A., Horak I.G., Latif A.A., Pegram R.G., Preston P.M. (2014). Ticks of Domestic Animals in Africa: A Guide to Identification of SPECIES.

[19] Khumalo C.S. (2024). Population Genetic Structure of Ixodid Ticks and Phylogenetic Analysis of Rickettsia in Chongwe and Chisamba Districts of Zambia, in School of Veterinary Medicine. Master’s Thesis.

[20] Chitanga S., Simulundu E., Simuunza M.C., Changula K., Qiu Y., Kajihara M., Nakao R., Syakalima M., Takada A., Mweene A.S. (2018). First molecular detection and genetic characterization of *Coxiella burnetii* in Zambian dogs and rodents. Parasit. Vectors.

[21] Martin M. (2011). Cutadapt removes adapter sequences from high-throughput sequencing reads. EMBnet. J..

[22] Li H. (2018). Minimap2: Pairwise alignment for nucleotide sequences. Bioinformatics.

[23] Li H., Handsaker B., Wysoker A., Fennell T., Ruan J., Homer N., Marth G., Abecasis G., Durbin R. (2009). The Sequence Alignment/Map format and SAMtools. Bioinformatics.

[24] Thompson J.D., Higgins D.G., Gibson T.J. (1994). CLUSTAL W: Improving the sensitivity of progressive multiple sequence alignment through sequence weighting, position-specific gap penalties and weight matrix choice. Nucleic Acids Res..

[25] Kumar S., Stecher G., Suleski M., Sanderford M., Sharma S., Tamura K. (2024). MEGA12: Molecular Evolutionary Genetic Analysis Version 12 for Adaptive and Green Computing. Mol. Biol. Evol..

[26] Nguyen S.V., Hirai K. (1999). Differentiation of *Coxiella burnetii* isolates by sequence determination and PCR-restriction fragment length polymorphism analysis of isocitrate dehydrogenase gene. FEMS Microbiol. Lett..

[27] Wyk C.V., Mtshali K., Taioe M.O., Terera S., Bakkes D., Ramatla T., Xuan X., Thekisoe O. (2022). Detection of Ticks and Tick-Borne Pathogens of Urban Stray Dogs in South Africa. Pathogens.

[28] Eneku W., Erima B., Byaruhanga A.M., Cleary N., Atim G., Tugume T., Ukuli Q.A., Kibuuka H., Mworozi E., Tweyongyere R. (2024). Molecular detection of *Coxiella burnetii* in ticks collected from animals and the environment in Uganda. Zoonoses Public Health.

[29] Kumsa B., Socolovschi C., Almeras L., Raoult D., Parola P. (2015). Occurrence and Genotyping of *Coxiella burnetii* in Ixodid Ticks in Oromia, Ethiopia. Am. J. Trop. Med. Hyg..

[30] Sulyok K.M., Hornok S., Abichu G., Erdélyi K., Gyuranecz M. (2014). Identification of novel *Coxiella burnetii* genotypes from Ethiopian ticks. PLoS ONE.

[31] Koka H., Sang R., Kutima H.L., Musila L. (2018). *Coxiella burnetii* Detected in Tick Samples from Pastoral Communities in Kenya. BioMed Res. Int..

[32] Ndeereh D., Muchemi G., Thaiyah A., Otiende M., Angelone-Alasaad S., Jowers M.J. (2017). Molecular survey of *Coxiella burnetii* in wildlife and ticks at wildlife-livestock interfaces in Kenya. Exp. Appl. Acarol..

[33] Reye A.L., Arinola O.G., Hubschen J.M., Muller C.P. (2012). Pathogen prevalence in ticks collected from the vegetation and livestock in Nigeria. Appl. Environ. Microbiol..

[34] Mediannikov O., Fenollar F., Socolovschi C., Diatta G., Bassene H., Molez J.F., Sokhna C., Trape J.F., Raoult D. (2010). *Coxiella burnetii* in humans and ticks in rural Senegal. PLoS Neglected Trop. Dis..

[35] Mtshali K., Khumalo Z., Nakao R., Grab D.J., Sugimoto C., Thekisoe O. (2015). Molecular detection of zoonotic tick-borne pathogens from ticks collected from ruminants in four South African provinces. J. Vet. Med. Sci..

[36] Smith T.A., Driscoll T., Gillespie J.J., Raghavan R. (2015). A Coxiella-like endosymbiont is a potential vitamin source for the Lone Star tick. Genome Biol. Evol..

[37] Brenner A.E., Muñoz-Leal S., Sachan M., Labruna M.B., Raghavan R. (2021). *Coxiella burnetii* and Related Tick Endosymbionts Evolved from Pathogenic Ancestors. Genome Biol. Evol..

[38] Halajian A., Palomar A.M., Portillo A., Heyne H., Romero L., Oteo J.A. (2018). Detection of zoonotic agents and a new Rickettsia strain in ticks from donkeys from South Africa: Implications for travel medicine. Travel. Med. Infect. Dis..

[39] Halajian A., Palomar A.M., Portillo A., Heyne H., Luus-Powell W.J., Oteo J.A. (2016). Investigation of Rickettsia, *Coxiella burnetii* and Bartonella in ticks from animals in South Africa. Ticks Tick Borne Dis..

[40] Guo H., Adjou Moumouni P.F., Thekisoe O., Gao Y., Liu M., Li J., Galon E.M., Efstratiou A., Wang G., Jirapattharasate C. (2019). Genetic characterization of tick-borne pathogens in ticks infesting cattle and sheep from three South African provinces. Ticks Tick Borne Dis..

[41] Varela-Castro L., Zuddas C., Ortega N., Serrano E., Salinas J., Castellà J., Castillo-Contreras R., Carvalho J., Lavín S., Mentaberre G. (2018). On the possible role of ticks in the eco-epidemiology of *Coxiella burnetii* in a Mediterranean ecosystem. Ticks Tick Borne Dis..

[42] González J., González M.G., Valcárcel F., Sánchez M., Martín-Hernández R., Tercero J.M., Olmeda A.S. (2020). Transstadial Transmission from Nymph to Adult of *Coxiella burnetii* by Naturally Infected Hyalomma lusitanicum. Pathogens.

[43] Derrick E.H. (1944). The epidemiology of Q fever. J. Hyg..

[44] Reedijk M., van Leuken J.P., van der Hoek W. (2013). Particulate matter strongly associated with human Q fever in The Netherlands: An ecological study. Epidemiol. Infect..

[45] Minnick M.F., Raghavan R. (2012). Developmental biology of *Coxiella burnetii*. Adv. Exp. Med. Biol..

[46] Sheek-Hussein M., Zewude A., Abdullahi A.S., Abdelgaleel N.H., Ishag H.Z.A., Yusof M.F., MS A.L., Shah A.M.A., AlNeyadi J., Osman B. (2025). One health approach based descriptive study on *Coxiella burnetii* infections in camels and abattoir workers in the United Arab Emirates. Sci. Rep..

[47] van Leuken J.P., Swart A.N., Droogers P., van Pul A., Heederik D., Havelaar A.H. (2016). Climate change effects on airborne pathogenic bioaerosol concentrations: A scenario analysis. Aerobiologia.

[48] Van Leuken J.P.G., Swart A.N., Brandsma J., Terink W., Van de Kassteele J., Droogers P., Sauter F., Havelaar A.H., Van der Hoek W. (2016). Human Q fever incidence is associated to spatiotemporal environmental conditions. One Health.

[49] van der Hoek W., Hunink J., Vellema P., Droogers P. (2011). Q fever in The Netherlands: The role of local environmental conditions. Int. J. Environ. Health Res..

