# Supplementary material for: Molecular Detection and Prevalence of Coxiella burnetii in Ticks from Namibia: A Regional and Genus-Specific Analysis
Source: Pathogens. 2025 Dec 10;14(12):1262. doi: 10.3390/pathogens14121262 (PMC12735502; doi:10.3390/pathogens14121262)
Supplement: Supplementary file 1 [file pathogens-14-01262-s001.zip › pathogens-3999260-supplementary.pdf]

Supplementary tables

S1: Table of sequence identity for tick samples used in this study.

	**12S rRNA**					**16S rRNA**				
**Sample ID**	**Blast result**	**Accession number**	**% Identity**	**Fragment length (bp)**	**Accession Number**	**Blast result**	**Accession number**	**% Identity**	**Fragment length (bp)**	**Accession Number**
Nam TD1121	*A. variegatum*	KY688458.1	100	344	PX443481	*A. variegatum*	LC634576.1	99.1	403	PX442698
Nam TD1146	*A. variegatum*	HQ856514.1	99.41	339	PX443482	*A. variegatum*	LC634546.1	99.26	403	PX442699
Nam TD1366	*A. variegatum*	HQ856466.1	100	340	PX443483	*A. variegatum*	PQ432625.1	99.49	391	PX442700
Nam TD1656	*A. variegatum*	AF150047.1	99.71	341	PX443484	*A. variegatum*	LC634588.1	98.27	405	PX442701
Nam TD1667	*A. variegatum*	HQ856542.1	100	340	PX443485	*A. variegatum*	MH781753.1	98.63	454	PX442702
Nam TD512	*H. rufipes*	KC817342.1	100	339	PX443476	*H. rufipes*	MK737650.1	100	407	PX442693
Nam TD788	*H. rufipes*	AF150033.1	100	341	PX443477	*H. rufipes*	KU130459.1	100	409	PX442694
Nam TD991	*H. rufipes*	KX000618.1	99.12	341	PX443478	*H. rufipes*	KU130463.1	100	409	PX442695
Nam TD1109	*H. rufipes*	AF031856.1	99.37	321	PX443479	*H. rufipes*	MK737650.1	100	407	PX442696
Nam TD1267	*H. truncatum*	AF150031.1	99.71	341	PX443480	*H. truncatum*	KU130478.1	99.5	409	PX442697
Nam TD1367	*R. evertsi evertsi*	AF150052.1	99.71	342	PX443472	*R. evertsi evertsi*	KJ613642.1	100	401	PX442689
Nam TD1299	*R. evertsi evertsi*	MZ351127.1	99.18	387	PX443473	*R. evertsi evertsi*	LC634571.1	99	400	PX442690

Nam TD482	*R. sanguineus*	JQ425164.1	96.38	383	PX443474	*R. sanguineus*	KX632154.1	99.53	428	PX442691
Nam TD1034	*R. evertsi mimeticus*	AF031862.1	100	321	PX443475	*R. evertsi mimeticus*	PP835222.1	100	395	PX442692
